# Impact of immunohistochemical profile changes following neoadjuvant therapy in the treatment of breast cancer

**DOI:** 10.3332/ecancer.2021.1162

**Published:** 2021-01-05

**Authors:** Gabriela Candás, Alejandra García, María Delfina Ocampo, Ernesto Korbenfeld, H. Daniel Vuoto, Juan Isetta, Lucas Cogorno, Agustina González Zimmermann, Marca Sigal, Santiago Acevedo, Julia Berwart, Martín Naveira, Agustina Bemi, Juan Luis Uriburu

**Affiliations:** 1 Breast Care Service, Buenos Aires British Hospital, Buenos Aires C1280AEB, Argentina; 2 Oncology Service, Buenos Aires British Hospital, Buenos Aires C1280AEB, Argentina; 3 Head of the Mastology Service, Buenos Aires British Hospital, Buenos Aires C1280AEB, Argentina

**Keywords:** neoadjuvant, hormonal receptors, HER2, variability, pathological complete response, adjuvant

## Abstract

**Introduction:**

Currently, the indication for neoadjuvant chemotherapy is increasing in the treatment of breast cancer. Variability in the expression of biomarkers following neoadjuvant treatment has been observed, which could be accompanied by changes in the adjuvant treatment.

**Objectives:**

The primary objective was to evaluate the variability of biomarkers prior to and following neoadjuvant therapy. Secondary objectives were to determine which tumour subtype (as determined by immunohistochemical markers) most frequently achieved pathological complete response (pCR); whether the biomarker variation resulted in a change in immunophenotype and subsequently modification to the adjuvant treatment.

**Materials and methods:**

A retrospective observational analysis was carried out on patients with a diagnosis of breast cancer who had neoadjuvant therapy prior to surgery in the Breast Care Service of the Buenos Aires British Hospital between January 2009 and June 2020.

**Results:**

One hundred and seventy-two patients were included. The pCR rate was 28.5%. The tumour immunophenotype that achieved pCR most frequently was the hormone receptor negative /HER2+ group with a value of 85.2%. The analysis was carried out on the 123 patients with residual disease. The observed variability for oestrogen receptors (ER) was 8.9%, for progesterone receptors (PR), 29.9% and for HER2, 13.8%. These changes were statistically significant. There were changes to the tumour immunophenotype in 26 cases (21.1%) with modifications to the adjuvant treatment in nine of these (34.6%; 7.3% of all tumours with residual disease).

**Conclusions:**

In this study, we observed statistically significant variability in the expression of ER, PR and HER2 prior to and following neoadjuvant treatment, which identified modifications in the tumour immunophenotype in 21.1%, and changes to the adjuvant treatment in 7.3% of all tumours with residual disease, justifying the re-assay of biomarkers in the surgical specimen.

## Introduction

Neoadjuvant chemotherapy (CTx) is an integral part of breast cancer treatment. Today, it is used ever more frequently in initial stages, particularly in the HER2+ and triple negative (TN) subgroups. It allows for an increase in the rate of conservative surgeries, values the live response to treatment, optimises adjuvant treatments when pathological complete response (pCR) is not achieved and increases disease-free survival (DFS) in those who do achieve pCR [1–3].

The best rates of pCR are obtained in the hormone receptor negative/HER2+ (HR-/HER2+) and TN subtypes, correlated with a better prognosis in terms of both DFS and overall survival [4]. On the contrary, the relationship between the rate of pCR and DFS is lower in luminal tumours.

In cases with residual disease, several studies report discordant results between the pre- and post-neoadjuvant treatment biomarkers, 15% for oestrogen receptors (ER), 30% for progesterone receptors (PR) and 10% for HER2+ cases [5, 6]. There are different explanations for this variation such as the heterogeneity of the tumour, technical issues, elimination of dominant clones due to treatment and the residual plasticity of the tumour due to the effect of the established therapy [7]. The lack of evidence in the literature on how changes in immunophenotype impacts the adjuvant treatments generates uncertainty in the management of these patients. However, different groups recommend repeating the biomarker assay with the aim of optimising the selection of adjuvant treatments, thereby maximising their benefits and reducing their toxicity.

This is why we decided to carry out this study—to evaluate the change in immunophenotype and its impact on treatment.

## Objectives

The main objective was to evaluate variations in the immunohistochemical (IHC) profile between the needle biopsy prior to neoadjuvant therapy and the surgical biopsy following neoadjuvant therapy.

Secondary objectives were proposed to analyse the pCR rate according to the tumour’s immunophenotype, analyse whether biomarker variations impacted the immunophenotype and evaluate the changes in adjuvant treatment.

## Materials and methods

A retrospective, descriptive, cross-sectional analysis was carried out on patients with a diagnosis of breast cancer. All of these patients underwent neoadjuvant treatment, and completed surgery with an indication for subsequent adjuvant therapy in the Breast Care Service of the Buenos Aires British Hospital between January 2009 and June 2020. The data from the medical records and the service database were analysed, having previously obtained authorisation from the Ethics Committee and the Institutional Review Board of the British Hospital (CRIHB#998). An exemption from the Ethics Committee was obtained for the need to obtain informed consent, given the retrospective nature of the study.

The following inclusion criteria were searched for the period from January 2009 to June 2020 in the Buenos Aires British Hospital: patients who underwent neoadjuvant treatment followed by surgery, and who had indication for subsequent adjuvant therapy. The exclusion criteria were metastatic disease when starting neoadjuvant therapy, and incomplete data in the medical records entry.

Medical records of 204 patients who intended to undergo neoadjuvant treatment in the British Hospital during this period were reviewed. Of these, 32 cases were excluded: 5 patients were excluded due to presentation with metastatic disease at the time of diagnosis and 27 due to incomplete data. The study comprised 172 patients who underwent neoadjuvant treatment and completed their surgeries with indication for subsequent adjuvant treatment.

The variables analysed were: age upon diagnosis, histological type, clinical staging (cTNM 8th edition) [8], IHC profile and their changes subsequent to neoadjuvant therapy, pathological staging following neoadjuvant therapy (ypTNM), type of surgery carried out, type of response obtained, adjuvant treatment and whether there were changes in this treatment due to the IHC profile variations.

The patients were evaluated by physical exam, mammogram, ultrasound and breast MRI with and without gadolinium contrast (pre- and post-systemic treatment), and histological diagnosis by core needle biopsy, vacuum assisted or punch biopsy.

HR was evaluated using immunohistochemistry and was considered positive for values of ≥1% in accordance with the guidelines from the American College of Pathologists [9]. HER2 was evaluated using IHC, considering (0/+) as negative, (++) as borderline and (+++) as positive. Borderline cases were re-evaluated using the FISH technique [10]. There was no central review for the needle and surgical biopsies.

The following tumour immunophenotypes were considered according to IHC: (1). Luminals in which HR were expressed with absence of over-expression of HER2 (HR+/HER2−); (2). Luminal HER2+ in cases which expressed HR and HER2 (HR+/HER2+); (3). HER2+ in those which only expressed the HER2 protein (RH−/HER2+) and (4). Triple negative (TN) if neither HR nor HER2 were expressed [11].

pCR was considered as the absence of invasive carcinoma in the breast tissue and the absence of compromised lymph nodes (ypT0N0). The presence of an in situ component in the breast tissue was accepted (ypTisN0) in accordance with the TNM staging system (8th edition) [8].

Regarding the statistical analysis, the qualitative variables are described as percentages; the quantitatives as median and range. The discordance in the pre- and post-neoadjuvant therapy biomarker results was analysed by the McNemar test. A value of *p* < 0.05 was considered to be statistically significant. The analysis was performed using the STATA 14.1 programme (StataCorp, Texas, USA).

## Results

One hundred and seventy-two patients who fulfilled the inclusion criteria for the study were evaluated. The median age on diagnosis was 51 years. The population analysed is described in Table 1. The most common characteristics prior to neoadjuvant treatment were: cT2 tumour 40.7%, cN+ (clinically positive axilla) 45.4%, clinical stage 2A 23.8%. Regarding the biopsy IHC, there were 119 ER+ cases (69.2%), 91 PR+ (52.9%) and 57 HER2+ cases (33.1%). 51.2% of the tumours corresponded to immunophenotype HR+/HER2−.

The majority of the patients received adriamycin + cyclophosphamide × 4 + taxanes × 12 (63.3%). The neoadjuvant treatments received are specified in Table 2, and in Table 3 they are described according to tumour immunophenotype.

### Pathological complete response

Forty-nine cases from the entire study population achieved pCR (28.5%). The analysis according to immunophenotype is shown in Table 4, the group which achieved the highest rate of pCR was HR−/HER2+ (85.2% of cases).

The 49 patients with pCR were excluded from the analysis of biomarker variability. The analysis was carried out on the 123 patients with residual disease.

### Immunohistochemistry

In the IHC study of the post-neoadjuvant therapy samples, there were 92 ER+ cases (74.8%), 31 ER− cases (25.2%), 54 PR+ cases (43.9%), 65 PR− cases (52.8%), 4 cases (3.3%) in which the PR were unable to be evauluated, 10 HER2+ cases (8.1%) and 113 HER2− cases (91.9%).

Regarding ER, there were 11 post-neoadjuvant treatment changes, becoming negative in ten cases (9.9%), and positive in only one case (4.5%). The difference was 8.9% and was statistically significant (*p* = 0.006).

Regarding PR, there were 35 changes, becoming negative in 29 cases (37.7%) and positive in 6 cases (15%). The difference was 29.9%, and was statistically significant (*p* < 0.0001). In six cases, the expression of PR was unable to be evaluated due to exhaustion of the needle biopsy or excised samples.

Regarding HER2, there were 17 changes, becoming negative in 15 patients (65.2%) and positive in 2 cases (2%). The discordance was 13.8%, and was statistically significant (*p* 0.001). This data is shown in Table 5.

### Tumour subtypes

In grouping the tumours according to immunophenotype, we observed that this had converted, in 26 (21.1%) of the 123 cases with residual disease, as a consequence of the changes in the biomarkers (Table 6).

Of the 82 luminal tumours (HR+/HER2−) which did not achieve pCR, four converted to TN, one to HR+/HER2+ and one to HR−/HER2+.

In the TN cases, 18 did not achieve pCR, but only one became luminal (HR+/HER2−).

Of the four HR−/HER2+ cases presenting with residual disease, one changed its immunophenotype to TN.

Finally, in the case of the 19 patients in the HR+/HER2+ subgroup, 3 converted to HR−/HER2+, 13 to HR+/HER2− and 2 to TN.

### Adjuvant treatment

The adjuvant treatments carried out are detailed in Table 7.

The adjuvant treatment was modified in 9 (34.6%) of the 26 patients who had changes in their immunophenotype; of the total of 123 patients with residual disease it represented 7.3% (Table 8).

In the 17 patients whose immunophenotype changed, there was no change in the recommended adjuvant treatment, as the patients who initially had HR or were HER2 positive did not have their adjuvant treatment altered when any of them became negative.

The 13 HR+/HER2+ patients whose immunophenotype changed to HR+/HER2− continued adjuvant therapy with hormone therapy (HT) and completed the course of trastuzumab.

The HR+/HER2+ patient whose post-neoadjuvant therapy biomarkers converted to negative, becoming TN, continued after surgery with HT and trastuzumab (underwent surgery prior to 2015).

Two of the HR+/HER2+ patients who became HR−/HER2+, continued with HT and completed the course of trastuzumab as adjuvant therapy. Further, a HR+/HER2+ patient who underwent surgery in 2020 and whose immunophenotype changed to HR−/HER2+ completed a course of adjuvant therapy with T-DM1 as well as HT.

In nine patients, the change in immunophenotype affected the adjuvant treatment.

The six patients whose immunophenotype changed to TN (four HR+/HER2−, one HR+/HER2+ and 1 HR−/HER2+), with surgeries after December of 2015, received CTx with capecitabine as adjuvant treatment and the five patients who were originally HR+ continued with HT. The two patients with HER2+ did not continue with anti-HER2 treatment.

In the three cases in which some of the biomarkers converted to positive, the adjuvant treatment was adjusted. Two patients were initially HR+/HER2− and converted to HR+/HER2+; treatment with tratuzumab was added to HT. The other patient with TN immunophenotype in which the HR converted to positive received HT as an adjuvant treatment.

## Discussion

Neoadjuvant therapy can be used as an initial treatment for breast cancer with the aim of reducing the tumour load, increasing the rate of conservative surgery, finding out the chemosensitivity of the tumour and, from the response achieved, obtaining information about the prognosis of the disease, effecting possible changes in the adjuvant treatment and prolonging the DFS in certain tumour subgroups [4].

The decision to carry out neoadjuvant therapy and the choice of the medication used are based on the biomarkers obtained from the needle biopsy (ER, PR and HER2). Different authors evidence the strong concordance between the data obtained in the needle biopsy and the first surgery [12, 13]. For this reason, treatment can be based on the diagnosis of the subgroup carried out by this method.

In our study, there were 49 cases (28.5%) with pCR. This agrees with the data published in the bibliography, in which variable figures ranging from 9% and 43% are reported [4, 14–16].

On analysing the pCR according to immunophenotype, we observed that it was most frequently achieved in HR−/HER2+ patients (85.2% of cases)—even higher than that published by the Memorial Sloan Kettering Cancer Center group, who report 59% [17], and American College of Surgeons Oncology Group (ACOSOG) Z1071, who report 53.6% [18]. The high rate of pCR in our group is probably due to the low number of HR−/HER2+ cases (*n* = 27), and that 21 (77.8%) of these patients were treated with a dual blockade, with similar results to those published in the KRISTINE study [19].

With respect to the variability in the biomarkers (ER, PR, HER2), different studies have been published, both retrospective and prospective, using different neoadjuvant therapy regimes and producing discordant results (Table 9).

Regarding the variation of the expression of the ER in the meta-analysis of Van de Ven *et al* [7], variable results have been reported in the different studies included. Those with the greatest number of patients presented statistically significant changes, with conversion to negative or reduction of ER expression predominating, as with other more recent retrospective series [20–22]. However, trials with smaller samples have not found significant changes. Penault-Llorca *et al* [1] analyse the loss of ER expression as a possible resistance mechanism for the tumour. Zhang *et al* [6] in their meta-analysis record a variability of 18.1%, this being statistically significant. Conversely, Jabbour *et al* [5] report an ER variability of 12.9%, with a tendency towards becoming negative. In our work we have a variability in the ER expression of 8.9%, being statistically significant, with the greatest number of cases converting to negative.

In relation to the PR, Van de Ven *et al* [7], Zhang *et al* [6] and Gahlaut *et al* [20] , Peng *et al* [21] and Yang *et al* [22] report statistically significant changes (14.5% to 70% of the cases), with a predominant tendency towards conversion to negative. Jabbour *et al* [5] report similar results with a variability of 32%, similar to Wu et al. with 26.9% [23]. In our series, the PR were those which presented with the greatest variability 29.9%; this was statistically significant, with a tendency towards conversion to negative of 24.8%.

With respect to the modification of HER2, the different retrospective studies and meta-analyses produce discordant results depending upon whether or not anti-HER2 therapy is used. Van de Ven *et al* [7], using HER2 blockade, reported between 32% and 42% loss of amplification of HER2. Pernault-Llorca *et al* [1] report variations with a predominant loss of HER2 in up to 43% of the cases with use of trastuzumab. In contrast, Jabbour *et al* [5] and Zhang *et al* [6], who do not include studies using HER2 blockades as a neoadjuvant treatment, report changes of 8.9% and 5.4%, respectively. These data do not achieve statistical significance. In our series we noted a statistically significant variability for HER2 of 13.8%, with a tendency towards conversion to negative, in agreement with those series using HER2 blockade therapies.

Several hypotheses are proposed as an explanation for this variability in the biomarkers: the tumours’ heterogeneity which could be not represented in the needle biopsy; technical problems or errors in the assays; the selection of clones resistant to systemic treatment following neoadjuvant therapy; changes in biomarker expression as a resistance mechanism of the tumour; a regression in the cellular differentiation of HR expression as a consequence of CTx or a negative regulation in the expression of HR due to ovarian suppression induced by CTx [7].

On evaluating whether the changes in the tumour biomarkers translated into immunophenotype alterations, De la Cruz *et al* [14] report subtype changes in 16.7% of the patients, similar to that observed in our series (21.1%). Although in the majority of cases, there was an observed tendency for the biomarkers to convert to negative following neoadjuvant therapy, we found opposing results given for retrospective studies in the literature (with potential greater biases). Lim *et al* [24] report changes in 23.1% of cases: the most frequently observed changes were from TN to luminal and the gain of hormonal receptors in HER2+; all these changes were statistically significant. Peng *et al* [21] describe that after neoadjuvant CTx, luminal A, HER2+ and TN tumours increase, whilst luminal B tumours decrease in a statistically significant fashion. What we observed most frequently in our work was the loss of HER2.

As to whether these changes derive from modifications to the adjuvant treatment, De la Cruz *et al* [14] report changes in the adjuvant treatment in all cases in which there were variations in the biomarkers (five patients). This is due to adding medication when a biomarker converted to positive in the hope of deriving a therapeutic benefit. Conversely, in cases of conversion to negative, medication was suspended with the aim of improving the patients’ quality of life.

Other works refer specifically to the change in the HR. In cases wherein receptors converted to positive, there is a consensus on the addition of adjuvant HT. However, in the case of conversion to negative, Chen *et al* [25] maintain that HT would not have a clear benefit. Meanwhile, Wu *et al* [26] agree that adjuvant HT in tumours which convert to negative in their HR expression had a statistically significant benefit in DFS, though not in global survival. The potential benefit sought with continuing endocrine treatment once the HR convert to negative following neoadjuvant treatment is treating the hormone-dependent micrometastatic tumour clones and/or chemoprevention in the contralateral breast.

Regarding the adjuvant treatment modifications for the HER2 variation, several publications recommend adding anti-HER2 treatment in cases of HER2 gain. On the other hand, in the cases of loss of HER2, the suggestion would be to continue with anti-HER2 agents considering the concept of tumour heterogeneity, but more evidence is required in this respect [27].

In agreement with this, two literature reviews state that, due to the high tumour heterogeneity observed, it could be possible that the changes in the biomarkers prior to and following neoadjuvant treatment could be due to differences in the samples, in the selection of clones or selective expression of different biomarkers, which suggest that despite a biomarker converting to negative, the indication would be to continue the corresponding adjuvant treatment [1, 28].

In our series, there were changes in adjuvant treatment in 9 of the 26 cases with altered immunophenotype (34.6%). In cases in which a biomarker converted to positive, it was decided to add specific medication, but it was not stopped when one converted to negative. Those which converted to TN after the CREATE-X study was presented in San Antonio in 2015 [2, 29], the adjuvant capecitabine was added.

Currently, HR+/HER2+ or HR−/HER2+ patients with residual disease following neoadjuvant treatment should be treated with adjuvant T-DM1 therapy. This is based on the favourable results from the KATHERINE study, which demonstrated the advantage of DFS in patients treated with T-DM1 versus patients treated with trastuzumab [3]. In our series, three patients received this treatment who were treated following approval of this drug.

The tumour biology is the most relevant factor today for those who make therapeutic decisions and this, together with the development of new drugs and target therapies, will offer greater curative possibilities in the future for these patient groups.

This matter has not yet been addressed by international guidelines [National Comprehensive Cancer Network (NCCN), European society for Medical Oncology (ESMO), St. Gallen] perhaps because it is an infrequent scenario. However, we consider it necessary to know the biomarker status of the residual disease following neoadjuvant treatment, even though the data from the literature may be discordant regarding the impact of the indicated treatments. Undoubtedly, these are challenging situations which will require evaluation and discussion within the multidisciplinary team.

## Conclusions

- The pCR was 28.5%, reaching a rate of 85.2% in the HR−/HER2+ subtype.
- The variation in biomarker expression of the residual disease was statistically significant: ER 8.9%, PR 29.9% and HER2 13.8%.
- The immunophenotype changed in 26 cases (21.1%). This translated to alteration of the adjuvant treatment in nine cases (34.6%) of those whose immunophenotype changed and 7.3% of all tumours with residual disease.
- We believe it is important to know the immunophenotype of the residual disease following neoadjuvant therapy since tumour biology is, today, the most relevant factor in therapeutic decisions. This, together with the development of new drugs and target therapies will offer greater possibilities for these patient groups in the future.

## Conflicts of interest

The authors have declared no conflicts of interest in this work.

## Funding

The authors have not received any financing to carry out this work.

## Figures and Tables

**Table 1.** Characteristics of the patients.

*n*	**172**	
*Age* *Median (range)*	**51 (24–77)**	
*T*	*n*	*Percentage*
1	36	20.9
2	70	40.7
3	49	28.5
4	17	9.9
*N*	*n*	*Percentage*
0	84	48.8
1	78	45.3
2	10	5.8
*Clinical stage*	*n*	*Percentage*
01AA	28	16.3
02AA	41	23.8
02BB	43	25.0
03AA	43	25.0
03BB	16	9.3
03CC	1	0.6
*Needle biopsy histology*	*n*	*Percentage*
NST	147	85.5
ILC	19	11.0
Mucinous	1	0.6
Micropapillary	1	0.6
Apocrine	3	1.7
Undifferentiated	1	0.6
*Needle biopsy IHC*	*n*	*Percentage*
Oestrogen receptors		
ER+	119	69.2
ER−	53	30.8
Progesterone receptors		
PR+	91	52.9
PR−	79	45.9
PR NE	2	1.2
HER2		
HER2+	57	33.1
HER2−	115	66.9
*Immunophenotypes*	*n*	*Percentage*
HR+/HER2−	88	51.2
HR−/HER2+	27	15.7
HR+/HER2+	31	18
TN	26	15.1

*n*, Number; NST, Invasive carcinoma of no special type; ILC, Invasive lobular carcinoma; IHC, Immunohistochemical; ER, Oestrogen receptors; PR, Progesterone receptors; HR, Hormone receptors; TN, Triple negative; NE, Not evaluable

**Table 2.** Neoadjuvant schemes used.

	*n* (%)
AC × 4 + paclitaxel × 12	109 (63.3%)
AC × 4 + paclitaxel × 12 + trastuzumab + pertuzumab	42 (24.4%)
AC × 4 + paclitaxel × 12 + trastuzumab	12 (7%)
AC × 4	6 (3.5%)
Carboplatin + docetaxel × 6	1 (0.6%)
AC × 4 + trastuzumab	1 (0.6%)
AC × 4 + trastuzumab + pertuzumab	1 (0.6%)

AC, Adriamycin + cyclophosphamide

**Table 3.** Neoadjuvant regimes according to immunophenotype.

Immunophenotype	Neoadjuvant therapy	*n* (%)
HR+/HER2−	AC × 4	4 (4.5%)
	AC × 4 + paclitaxel × 12	84 (95.5%)
HR−/HER2+	AC × 4 + trastuzumab + pertuzumab	1 (3.7%)
	AC × 4 + paclitaxel × 12 + trastuzumab	5 (18.5%)
	AC × 4 + paclitaxel × 12 + trastuzumab + pertuzumab	21 (77.8%)
HR+/HER2+	AC × 4	1 (3.2%)
	AC × 4 + trastuzumab	1 (3.2%)
	AC × 4 + paclitaxel × 12	1 (3.2%)
	AC × 4 + paclitaxel × 12 + trastuzumab	7 (22.6%)
	AC × 4 + paclitaxel × 12 + trastuzumab + pertuzumab	21 (67.7%)
TN	AC × 4	1 (3.8%)
	AC × 4 + paclitaxel × 12	24 (92.3%)
	Carboplatin + Docetaxel × 6	1 (3.8%)

AC, Adriamycin + cyclophosphamide

**Table 4.** pCR according to immunophenotype.

	*n* total	pCR
Total	172	49 (28.5%)
HR+/HER2-	88	6 (6.8%)
HR−/HER2+	27	23 (85.2%)
HR+/HER2+	31	12 (38.7%)
TN	26	8 (30.8%)

pCR, Pathological complete response; TN, Triple negative; HR, Hormone receptors

**Table 5.** Tumour biomarkers prior to and following neoadjuvant therapy (*n*: 123).

	Total (−) pre-neo	(−) >> (−)	(−) >> (+)	Total (+) pre-neo	(+) >> (+)	(+) >> (−)	Δ	p*
ER	22	21 (95.5%)	1 (4.5%)	101	91 (91.1%)	10 (9.9%)	11 (8.9%)	0.006
PR	40	34 (85%)	6 (15%)	77	48 (62.3%)	29 (37.7%)	35 (29.9%)	0.0001
HER2	100	98 (98%)	2 (2%)	23	8 (34.8%)	15 (65.2%)	17 (13.8%)	0.001

Δ, Variability; ER, Oestrogen receptors; PR, Progesterone receptors; Pre-neo, Prior to neoadjuvant therapy

* The *p* value was calculated using the McNemar test

**Table 6.** Variability in immunophenotype (*n*: 123).

		Post-neoadjuvant therapy			
Pre-neo	Total	HR+/HER2−	TN	HR−/HER2+	HR+/HER2+
HR+/HER2−	82	76 (92.7%)	4 (4.9%)	1 (1.2%)	1 (1.2%)
HR−/HER2+	4	-	1 (25%)	3 (75%)	-
HR+/HER2+	19	13 (68.4%)	2 (10.5%)	3 (15.8%)	1 (5.3%)
TN	18	1 (5.6%)	17 (94.4%)	-	-

TN, Triple negative; HR, Hormone receptors; Pre-neo, Prior to neoadjuvant therapy

**Table 7.** Adjuvant treatment regimes used.

HT	68 (55.3%)
HT + anti-HER2	19 (15.5%)
None	13 (10.6%)
CTx	10 (8.1%)
CTx + HT	9 (7.3%)
CTx + HT + anti-HER2	2 (1.6%)
Anti-HER2	2 (1.6%)

HT: hormone therapy; CTx: chemotherapy

**Table 8.** Adjuvant treatments in patients whose immunophenotype changed.

Pre-neo	Post-neo	*n*	Adjuvant therapy
**17 patients with change in immunophenotype without change in treatment**			
HR+HER2+	HR+HER2−	13	HT + trastuzumab
HR+HER2+	TN	1	HT + trastuzumab^a^
HR+HER2+	HR−HER2+	2	HT + trastuzumab
HR+HER2+	HR−HER2+	1	HT + T-DM1^b^
**9 patients with change in immunophenotype and change in treatment**			
HR+HER2-	TN	4	capecitabine + HT
HR+HER2+	TN	1	capecitabine + HT
HR−HER2+	TN	1	capecitabine
HR+HER2-	HR+HER2+	2	HT + trastuzumab
TN	HR+HER2−	1	HT

Pre-Neo, Immunophenotype prior to neoadjuvant therapy; Post-Neo, Immunophenotype following neoadjuvant therapy; HR, Hormone receptors; TN, Triple negative; HT, Hormone therapy

a patient previously operated on in 2015,

b patient operated on in 2020

**Table 9.** Studies describing biomarker variability.

Study	*n*	Neoadjuvant therapy	ER variability	PR variability	HER2 variability
Gahlaut (2016) [20]	246	CTx + Anti-HER2	12%	14.5%	7.1%
Yang (2018) [22]	231	CTx	5.6%	19.5%	NS
Peng (2018) [21]	112	CTx	22.3%	28.6%	20.5%
Wu (2018) [23]	525	CTx	15.2%	26.9%	NE
The present study (2020)	172	CTx + Anti-HER2	8.9%	29.9%	13.8%

CTx, Chemotherapy; ER, Oestrogen receptors; PR, Progesterone receptors; NE, Not evaluated; NS, Not significant
